# Potential Novel Risk Factor for Breast Cancer: *Toxocara canis* Infection Increases Tumor Size Due to Modulation of the Tumor Immune Microenvironment

**DOI:** 10.3389/fonc.2020.00736

**Published:** 2020-05-29

**Authors:** Rocío Alejandra Ruiz-Manzano, Margarita Isabel Palacios-Arreola, Rosalía Hernández-Cervantes, Víctor Hugo Del Río-Araiza, Karen Elizabeth Nava-Castro, Pedro Ostoa-Saloma, Samira Muñoz-Cruz, Jorge Morales-Montor

**Affiliations:** ^1^Departamento de Inmunología, Instituto de Investigaciones Biomédicas, Universidad Nacional Autónoma de México, Ciudad de México, Mexico; ^2^Departamento de Genotoxicología y Mutagénesis Ambiental, Centro de Ciencias de la Atmósfera, Universidad Nacional Autónoma de México, Ciudad de México, Mexico; ^3^Laboratorio de Inmunología y Biología Molecular de Parásitos, Facultad de Medicina Veterinaria y Zootecnia, Departamento de Parasitología, Universidad Nacional Autónoma de México, Ciudad de México, Mexico; ^4^Unidad de Investigación Médica en Enfermedades Infecciosas y Parasitarias, Instituto Mexicano del Seguro Social, Ciudad de México, Mexico

**Keywords:** oncoimmunology, immune regulation, tumor microenvironment, breast cancer, risk factor, infection

## Abstract

Worldwide, breast cancer is the most important type of cancer in women with regard to incidence and prevalence. Several risk factors interact to increase the probability of breast cancer development. Biological environmental contaminants such as infectious agents play a significant role in tumor development, and helminths have been recognized as cancer enhancers or inducers due to their ability to regulate the host immune response. *Toxocara canis* is a zoonotic and cosmopolite nematode with immuno-regulatory abilities. *T. canis* infection has been related to T helper type-2 cell (Th2 or type 2) and regulatory responses. Type 2 and regulatory immune responses may favor the development of comorbidities that are usually controlled or eliminated through a type 1 response such as cancer. The aim of this study was to determine whether *T. canis* infection alters mammary tumor growth through modulation of the immune response. Infected mice developed larger tumors. Tumor immune cell milieu analysis revealed that infection reduced the proportions of CD8^+^ lymphocytes and increased the proportions of F4/80^+^ macrophages and CD19^+^ B cells. These changes were accompanied by a type 2 local response represented by increased amounts of IL-4 and VEGF and a regulatory microenvironment associated with higher IL-10 levels. Thus, this study demonstrates that *T. canis* infection enhances tumor development and suggests that this is through modulation of the tumor immune microenvironment.

## Introduction

Breast cancer is the most prevalent cancer in women and their leading cause of cancer death (1). There are several risk factors associated with breast tumor development. First and foremost is gender; women present with breast cancer more frequently than men (1). Risk factors such as alcohol intake, smoking, and hormone replacement treatment, among others, have been linked to breast cancer development (2). Nevertheless, other environmental factors also play a prominent role in breast tumor development. Environmental contaminants, including physical, chemical, and biological agents, have been associated with tumor development (3). In fact, 15% of different cancer types are linked to viral, bacterial, or parasite infections (4). In this sense, some helminthic infections play an important role in cancer progression. The helminths are ubiquitous parasites that cause chronic infections in human, livestock, and domestic animals; they include platyhelminths (flatworms) and nematodes (roundworms) (5, 6). Most of the parasite species from the phylum Platyhelminths are cestodes (tapeworms) and trematodes (flukes), while the phylum Nematoda contains ascarides and strongylids among other roundworms (5, 6). For example, some trematodes are considered to cause bladder cancer (*Schistosoma haematobium*) and cholangiocarcinoma (*Chlonorchis sinensis* and *Opisthorchis viverrini*) (7, 8). Nematode infections are also reported as colon cancer enhancers (*Heligmosomoides polygyrus*) (9).

Helminths may promote tumor growth through different mechanisms that are related to chronic infection and long-lasting inflammation (7, 8). Chronic inflammation is mediated by helminth excretory-secretory (ES) products that modify the host immune response to the parasite and diminish tissue damage in the host (10). These processes allow helminth survival and may favor the development of other diseases.

In this regard, Treg expansion is stimulated by nematode infections (9, 11). It is known that Treg cells exert immune suppression through secretion of tolerogenic cytokines such as IL-10 and the dysfunction of cytotoxic T CD8^+^ cell activity (12, 13). Aside from Treg lymphocyte generation, nematodes also induce the generation of other regulatory cells and soluble factors associated with the promotion of tumor growth, thereby worsening prognosis by promoting metastasis (14). Among these are alternatively activated macrophages (AAMs), Breg lymphocytes, and IL-10 (10). AAMs are involved in wound healing and humoral response and produce IL-4, IL-10, VEGF, and other soluble factors (15, 16).

Macrophages are also linked to tumor development, as tumor-associated macrophages (TAMs). An AAM phenotype and a higher-density infiltration of these cells in breast tumors are associated with a worse prognosis (14). Another soluble factor produced by AAMs is VEGF, which is involved in angiogenesis promotion by increasing the sprouting and infiltration of new blood vessels in the tumor, leading to higher oxygen and nutrient levels, which enhance tumor cell proliferation (17).

The importance of helminth infection is not only related to the regulatory functions of these parasites but also to their rates of infection and their geographic distribution. A helminth that possesses immuno-regulatory properties and infects several hosts, including humans, worldwide is *Toxocara canis* (18).

*T. canis* has a wide range of hosts, including definitive (canids) and paratenic hosts such as humans, cats, lambs, pigs, cows, mice, rats, cockroaches, and flies (19–23). In paratenic hosts, *T. canis* larvae never develop into the adult form but migrate through different organs, including lungs, liver, heart, skeletal muscles, brain, and eyes (24). This migration induces a broad spectrum of signs and symptoms, which are characterized in humans as visceral, ocular, covert larva migrans, and neurotoxocariasis depending on the place where the larvae lodges and induces damage (24, 25). Although positive human sera to *T. canis* is reported worldwide, with serological frequencies in humans as high as 86.75% (26), an accurate incidence rate per country has not been established; therefore, national surveys are needed to determine the actual infection risk in humans due to the elevated rate of *T. canis*-infected dogs that excrete *T. canis* eggs in feces and contaminate the environment (27). Thus, the lack of information about the real incidence of this disease is hazardous *per se* because *T. canis* infection is an important neglected disease that could potentially affect the development of other pathologies.

The host immune response is induced by the *T. canis* larvae excretory-secretory (TES) products, and the evasion of this response allows the nematode to survive for many years in different host tissues (18). The mouse immune response to *T. canis* chronic infection has been reported as a type 2 response and a regulatory one (28). This is characterized by an increased proportion of F4/80^+^ macrophages, CD19^+^ lymphocytes, and CD4^+^Foxp3^+^ Treg cells in the spleen, as well as higher splenic and serum levels of IL-4, IL-10, and VEGF (28). For the abovementioned reasons, *T. canis* could regulate the host immune response, and in turn, favor tumor growth (29).

Consequently, we aimed to elucidate the role of *T. canis* infection in the development of mammary tumors and the associated local and systemic immune response.

## Methods

### Ethics Statement

The experimental procedures and animal care were performed at the Instituto de Investigaciones Biomédicas (IIB), Universidad Nacional Autónoma de México (UNAM), in the Biological Models Unit (Unidad de Modelos Biológicos, or UMB). These procedures were evaluated and approved by the Institutional Care and Animal Use Committee (CICUAL) (permit number 2017–208), in accordance with Mexican regulation (NOM-062-ZOO-1999) and with the Guide for the Care and Use of Laboratory Animals of the National Institute of Health (NIH) of the United States of America.

Blood samples were collected by cardiac puncture in deeply anesthetized animals (Sevofluorane 5%, Abbot, Mexico). Anesthetized mice were euthanized through cervical dislocation. Sera were obtained by blood centrifugation and were stored at −70°C until use.

### Animals

Twenty female mice, BALB/c AnN (MGI Cat# 5654849, RRID:MGI:5654849), 8–9 weeks old, were obtained from Envigo México (Facultad de Química, UNAM, México). They were maintained under standard conditions: controlled temperature (22°C), 12-h light-dark cycles, ad libitum water, and Envigo LabDiet 5015 (Cat# 0001328 Purina, St. Louis, MO) delivered in sterile conditions.

Mice were randomized into two experimental groups: 4T1 (tumor induction) and 4T1+*T. canis* (infection and tumor induction), each one with 10 animals. Infection was performed for 4T1+*T. canis*; meanwhile, the 4T1 group was administered phosphate-buffered saline solution (PBS, pH 7.4). Twenty-one days post-infection (dpi), a tumor was induced in both groups. Tumor growth was observed for 28 days.

### *Toxocara canis* Infection

Adult *T. canis* specimens were obtained from dog feces and washed 3 times with PBS and PBS/2% formaldehyde solution. The uteri were excised from adult *T. canis* females through cuticle incision in the anterior section. Eggs were extracted and filtrated through a fine mesh to eliminate debris, and then the suspension was centrifuged at 3250 g/5 min (HERMLE Z400K) and resuspended in PBS. The resulting suspension was maintained at room temperature. Larvae development was supervised every week.

When 80–90% of the eggs were larvated, the suspension was ready to induce the infection. This was performed in overnight fasted mice by administering 500 larvated eggs, which were inoculated *per os* with an oral feeding needle.

### Cell Culture and Mammary Tumor Induction

The 4T1 mammary mouse carcinoma cell line (ATCC Cat# CRL-2539, RRID:CVCL_0125) was grown in supplemented RMPI 1640 medium (Sigma, St. Louis, MO) with 10% FBS (ByProducts, Guadalajara, México), 1.0 mM sodium pyruvate, 100 U/ml penicillin, and 100 mg /ml streptomycin. The cells were harvested after a second subculture at 80% of confluency, resuspended in sterile 0.9% NaCl solution (250,000 cells/ml), and maintained in ice until inoculation.

The mice were anesthetized (Sevofluorane 5%, Abbot, Mexico), the abdominal area was aseptically prepared, and 10^4^ 4T1 cells were injected subcutaneously into the fat pad under the second last right nipple. Mouse recovery was supervised.

### *Toxocara canis* Larvae Cultures and Collection of TES Products

TES products were obtained based on methods by De Savigny (30) and Bowman (31), with modifications. Briefly, larvated egg suspension was centrifuged at 3250 g/5 min. To disaggregate the outer egg layer, 1 ml of sodium hypochlorite was added. After 10 min in continuous gentle agitation, the eggs were washed with 10 ml of bi-distillate sterile water, centrifuged at 3250 g/5 min, and washed three times with PBS. The eggs were resuspended in RPMI 1640 (Sigma, St. Louis, MO) with 1% antibiotic-antimycotic (GIBCO). Larva hatching was stimulated with a magnetic stirrer for 20 min, and the egg suspension was maintained at 37°C in a humidified atmosphere containing 5% (v/v) CO_2_ overnight. The larvae were cleaned from eggshells through a modified Baermann technique and maintained at a concentration of 10^4^ larvae/ml. The supernatant was recollected weekly and filtered with 0.22 μm syringe filter (Millipore); the medium was replaced. Protein was precipitated with acetone (Herschi Trading, high purity, 99.5%) at −20°C, resuspended in PBS, and stored at −20°C until use. Protein concentration was calculated with the Bradford Protein Assay Bio Rad® technique.

### Anti-*Toxocara canis* IgG Detection

Coated polystyrene wells (96-well plate, MaxiSorp Nunc Cat# NNC#442404) with 50 μl of TES/bicarbonate buffer (pH 9.6) suspension (1 μg/ml) were incubated at 4°C overnight. The plate was washed (PBS/Tween 20 0.05%) and blocked with 200 μl of 3% bovine serum albumin (BSA) and SIGMA washing solution for 30 min at 37°C. After the plate had been washed three times, 50 μl of sera from the mice was added (1:200 in PBS 1% BSA, 0.05%Tween 20) in duplicate and incubated for 1 h at room temperature. The plate was washed, and 50 μl of peroxidase goat anti-mouse IgG (Jackson, RRID:AB_2338511) at 1:10,000 dilution was added, followed by standing for 90 min at room temperature. An enzyme-substrate reaction was developed by the addition of 50 μl of freshly prepared substrate solution (0.05% o-phenylenediamine/0.01% H_2_O_2_/0.1 M sodium citrate/0.1 M citric acid) and stopped after 10 min with 50 μl 2N sulfuric acid. The plate was read at a wavelength of 492 nm in a Stat Fax 4200 microplate reader (Awareness Technology).

### Flow Cytometry

The left and right peripheral (inguinal) lymph nodes (PLNs) and the spleen were excised and mechanically disaggregated through a 50-μm nylon mesh with PBS. Tumors were excised and minced with a scalpel. After the PBS wash, the lymph node cells were resuspended in FACS buffer (PBS, 2% FBS, 0.02% NaN_3_). Erythrocytes in splenic suspension were lysed for 10 min with ACK buffer (150 mM NH_4_Cl, 10 mM KHCO_3_, 0.1 mM Na_2_ EDTA, pH 7.3), washed with PBS and resuspended in FACS buffer. The minced tumors were incubated in digestion medium (RPMI 1640, 10 U/ml DNase, Roche, Mannheim, Germany; 0.5 mg/ml type IV Collagenase, Sigma, St. Louis, MO) for 20 min, and 50 μl FBS was added to stop digestion. Mechanical disruption in a 50-μm nylon mesh was performed. After the PBS wash, the cells were resuspended in FACS buffer. Approximately 1 × 10^6^ cells were incubated (20 min at 4°C) with anti-CD16/CD32 (TruStain®, Cat# 101319, Clone 93, RRID:AB_1574973, BioLegend, San Diego, CA) and washed. Then, they were stained with the following panels. For T lymphocyte: AlexaFluor®488-conjugated anti-CD3ε (Cat# 100321, Clone 145-2C11, RRID:AB_389301) 1:100, PE-conjugated anti-CD4 (Cat# 100407, Clone GK1.5, RRID:AB_2075573) 1:300, PerCP-conjugated anti-CD8 (Cat# 100732, Clone 53-6.7, RRID:AB_893423) 1:100, and AlexaFluor®647-conjugated anti-Foxp3 (Cat# 320013, Clone 150D, RRID:AB_439750) 1:100. For macrophage and NK: AlexaFluor® 647-conjugated anti-F4/80 (Cat# 123122, Clone BM8, RRID:AB_893492) and PE-conjugated anti-NKp46 (Cat# 137604, Clone 29A1.4, RRID:AB_2235755). For B lymphocyte: PE-conjugated anti-CD19 (Cat# 115507, Clone 6D5, RRID:AB_313642), 1:200. Antibodies from BioLegend, San Diego, CA, and the Foxp3/Transcription Factor Staining Buffer kit (Cat# TNB-0607-KIT, Tonbo Biosciences, San Diego, CA) were used for intracellular Foxp3 staining, according to the manufacturer's protocol.

Cell analysis was performed with a BD FACSCalibur^TM^ (BD Biosciences) flow cytometer. The data were analyzed with FlowJo software (Treestar Inc.). Compensation was assessed in BD FACSCalibur^TM^ and FlowJo software with unstained samples, single stain controls, and FMO for Foxp3^+^ (CD3^+^/CD4^+^; CD3^+^/CD8^+^).

### Cytokine Determination

The tumors and spleens from the mice were stored in TRIzol^TM^ reagent (Cat# 15596026, Invitrogen) at −70°C until use. Protein isolation was performed according to the procedural guidelines for TRIzol® reagent use. Protein quantification was done with a NanoDrop 1000 spectrophotometer (Thermo Scientific). An amount of 10 μg of protein was used to determine cytokine tissue levels.

Sera, splenic, and tumor cytokines were measured with ABTS ELISA kits (PeproTech) with the following antibodies: TNF-α (Cat# 500-P64bt, RRID:AB_147984), IFN-γ (Cat# 500-P119bt, RRID:AB_148087), IL-4 (Cat# 500-P54bt, RRID:AB_147636), IL-5 (Cat# 500-P55), and IL-10 (Cat# 500-P60, RRID:AB_147978), and unconjugated antibodies were used for cytokine capture, according to the manufacturer's instructions, with modifications. Briefly, coated plates (96-well plate, MaxiSorp Nunc Cat# NNC#442404) with 50 μl (2 μg/ml) of different antibodies were incubated overnight. After 3 washes (wash buffer, PeproTech), the plates were blocked (block buffer: PeproTech) and then washed again. Next, 50 μl of sera (1:2 dilution) or tissue protein (10 μg) was added in duplicate (in diluent solution, PeproTech), maintained at 4°C for 2 h, and washed three times. An enzyme-substrate reaction was developed with ABTS liquid substrate (PeproTech). All solutions were from the ABTS ELISA buffer kit (Cat# 900-K00). The plates were read at a wavelength of 405 nm with wavelength correction set at 650 nm at different time points in a Stat Fax 4,200 microplate reader (Awareness Technology).

### VEGF Quantification

Polystyrene wells (96-well plate, MaxiSorp Nunc Cat# NNC#442404) were coated with 50 μl of splenic protein (10 μg), sera (dilution 1:2), or standard curve (0.001-1 ng) with VEGF mBA-165 (Cat# sc-4571, Santa Cruz Biotechnology) in bicarbonate buffer (pH 9.6) per duplicate and incubated at 4°C overnight. The plate was washed and blocked with 200 μl of PBS/BSA 1%/Tween 20 0.05% for 1 h at 4°C. After washing, 50 μl of anti-VEGF/C-1 antibody (Cat# sc-7269, RRID:AB_628430, Santa Cruz Biotechnology) in a 1:200 dilution was added, followed by incubation for 1 h at 4°C. After washing, 50 μl of m-IgGκ/BP-HRP (Cat# sc-516102, RRID:AB_2687626, Santa Cruz Biotechnology) (1:400) was added and maintained for 2 h at room temperature. An enzyme-substrate reaction was developed with 50 μl of substrate solution and stopped after 15 min with 50 μl 2N sulfuric acid. The plates were read at a wavelength of 492 nm in a Stat Fax 4,200 microplate reader (Awareness Technology). Cytokine and VEGF concentrations were calculated by interpolation from a standard curve.

Cytokine and antibody determination were performed after proper ELISA standardization.

### Statistical Analysis

Data were charted as mean ± SD. To compare the differences between intact and infected animals, a Student's *t*-test was used. A Welch's correction was applied in the groups in which the variances were different, as determined by an *F*-test. The differences were considered significant when *p* ≤ 0.05. All the analyses were calculated with Prism 6® software (GraphPad Sofware Inc.).

## Results

### *Toxocara canis* Infection Increases Tumor Size and Weight

**Figure 1:**
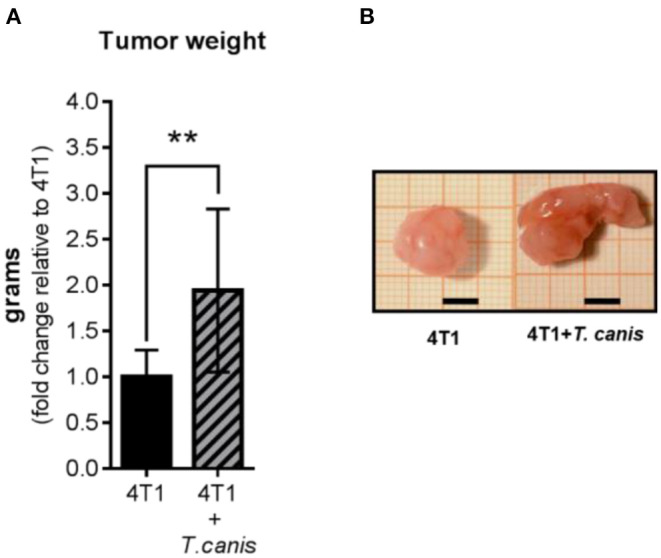
Tumor growth. **(A)** Tumor weight from control (4T1) and infected animals (4T1-*T. canis*). **(B)** Representative images of tumors from both groups (4T1 and 4T1+*T. canis*). Millimetric grid as a background; black bar = 0.5 cm. Graphs represent the mean ± SD of the data from two experiments (4T1, *n* = 10; 4T1+*T. canis, n* = 10). Statistical significance was calculated using a *t*-test (**p ≤ 0.01).

After 28 days of 4T1 cell inoculation, higher tumor enlargement was observed in *T. canis*-infected animals compared to tumors from the 4T1 mice group. This was quantified by measuring tumor mass, which was greater in the 4T1+*T. canis* group (*p* = 0.0035) (Figures 1A,B) than in the 4T1 group. In these infected mice, the mean tumor mass was almost doubled (91% increase) in mean weight compared to the group without infection.

### Increased Tumor Growth Is Associated With Changes in the Tumor Microenvironment

**Figure 2:**
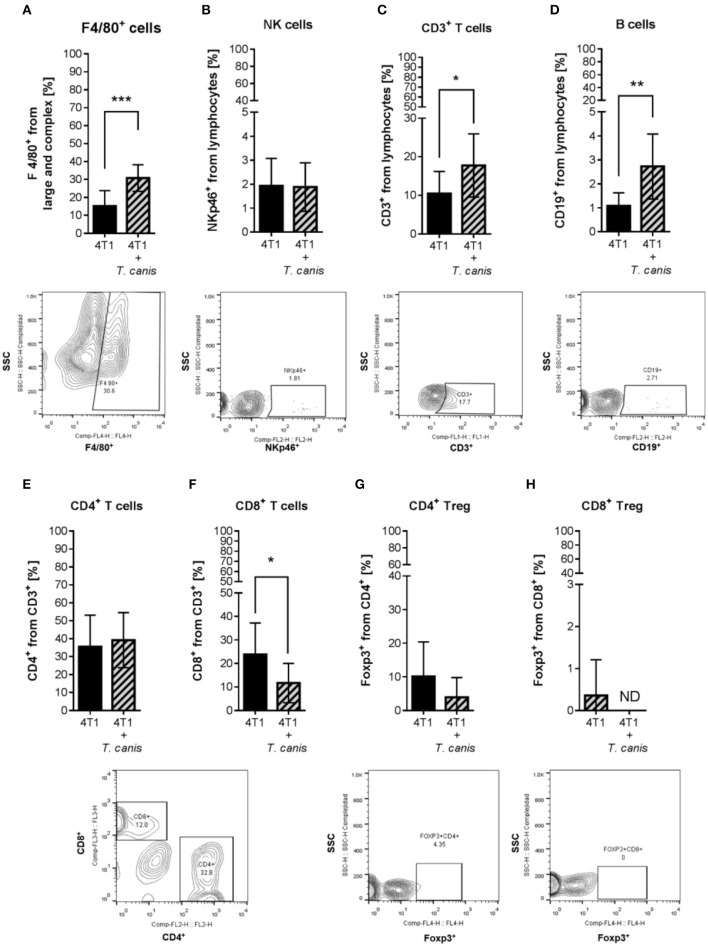
Innate and adaptive immune cells in the tumor microenvironment. Determination of innate immune subpopulations by flow cytometry. **(A)** F4/80^+^ macrophages. **(B)** NKp46^+^ cells. Flow cytometry analysis of adaptive immune cells within the tumor. **(C)** T CD3^+^. **(D)** CD19^+^ B lymphocytes. **(E)** CD4^+^ T helper lymphocytes. **(F)** CD8^+^ T cytotoxic lymphocytes. **(G)** CD4^+^/Foxp3^+^ T lymphocytes. **(H)** CD8^+^/Foxp3^+^ T lymphocytes. Graphs represent the mean ± SD of the data from two independent experiments (4T1, *n* = 10; 4T1+*T. canis, n* = 10). Representative contour plots of the cytometric analysis of the corresponding population (below). Gate from 10,000 cells was collected. Statistical significance was calculated using a *t*-test (*p ≤ 0.05; **p ≤ 0.01, ***p ≤ 0.001).

Because *T. canis* infection modifies the mouse's immune systemic response toward type 2 and regulatory responses (28), we wondered whether this change may reach the tumor microenvironment. To determine if tumor enlargement was associated with tumor microenvironment changes in innate (F4/80^+^ macrophages and NK cells) and adaptive (T, T helper, T cytotoxic, Treg, and B lymphocytes) cell proportions, we performed a flow cytometry evaluation. The percentage of F4/80^+^ macrophages was higher (*p* = 0.0004) in the 4T1+*T. canis* group (Figure 2A) compared to the 4T1 group. NK cells were present in a low percentage in tumors from both groups, and no change was observed in the proportions of this population (Figure 2B).

As for the adaptive populations in the tumor milieu, statistically, differences were observed in T lymphocyte CD3^+^, B cells CD19^+^, and T cytotoxic CD8^+^, but not in T helper (CD4^+^) or Treg (Figure 2) cells. In *T. canis*-infected mice, there were increased proportions of CD3^+^ (*p* = 0.0334) and CD19^+^ (*p* = 0.0042) lymphocytes (Figures 2C,D) and a decreased percentage of CD8^+^ cells (*p* = 0.0279) in the tumors (Figure 2F).

**Figure 3:**
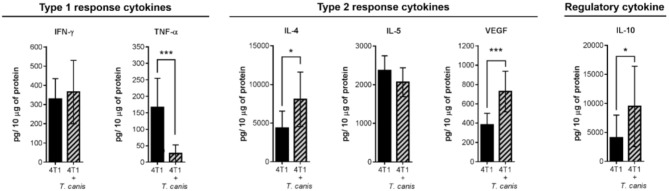
Expression of soluble factors in the tumor microenvironment. Analysis of type 1 (IFN-γ and TNF-α), type 2 (IL-4, IL-5, and VEGF), and regulatory (IL-10) cytokines. Graphs indicate data from tumor proteins obtained from two independent experiments (4T1, *n* = 10; 4T1+*T. canis, n* = 10). Bars represent the mean ± SD of cytokine levels (pg/10 μg of tumor protein). Statistical significance was calculated using a *t*-test (*p ≤ 0.05; ***p ≤ 0.001).

On the other hand, soluble factors in the tumor microenvironment are also important drivers of the local immune response. Therefore, we determined tumor cytokine expression of type 1 (TNF-α and IFN-γ), type 2 (IL-4 and IL-5), and regulatory (IL-10) cytokines. In the present study, the cytokine tumor milieu from 4T1+*T. canis* is polarized toward a type 2 and regulatory response. This is evidenced by the reduced amount of TNF-α (*p* = 0.0006) and the higher amounts of IL-4 (*p* = 0.0111), VEGF (*p* = 0.0003), and IL-10 (*p* = 0.0456) (Figure 3).

### Systemic Humoral *T. canis* Response Is Preserved in Tumor-Bearing Mice

**Figure 4:**
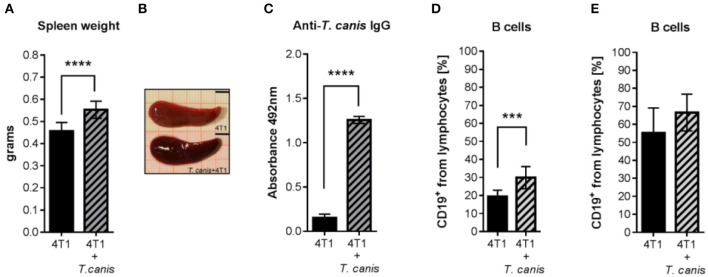
Humoral response to *T. canis* infection in BALB/c mice. **(A)** Splenic weight. **(B)** Representative images of a spleen from a mouse with tumors (4T1 group) and of a spleen from a mouse with tumors and infection (4T1+*T. canis*). Millimetric grid as a background. Black bar = 0.5 cm. **(C)** Anti-*T. canis* IgG serum levels measured by indirect ELISA. **(D)** Frequencies of CD19^+^ B lymphocytes in the spleen. **(E)**. Frequencies of CD19^+^ B lymphocytes in PLNs. Graphs represent the mean ± SD of the data from two experiments (4T1, *n* = 10; 4T1+*T. canis, n* = 10). Statistical significance was calculated using a *t*-test (***p ≤ 0.001; ****p < 0.0001).

Characteristic *T. canis* infection-associated splenomegaly was detected in the 4T1+*T. canis* group (Figures 4A,B). In these mice, the splenic weight was higher than the splenic weight in the control animals from the 4T1 group (*p* < 0.0001) (Figure 4A). In addition, the anti-*T. canis* IgG level (Figure 4C) was higher in infected mice than in tumor-bearing uninfected mice (*p* < 0.0001). There was an increased percentage of B cells in the spleen (*p* = 0.0002), but in the lymph nodes, there was no statistically significant increase in this subpopulation (Figures 4D,E).

### Systemic Immune Response to *T. canis* Is Modified by Tumor Induction

**Figure 5:**
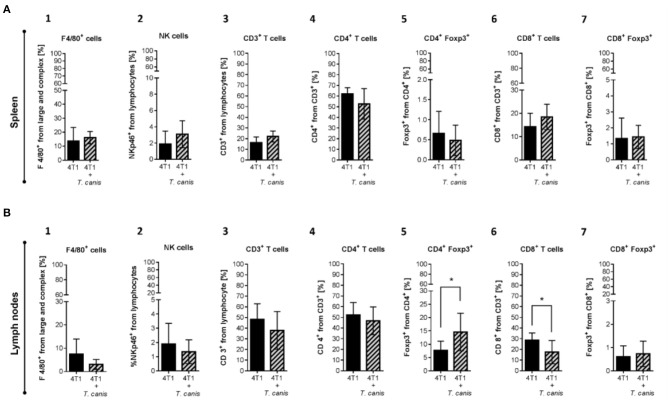
Innate and adaptive immune cells in secondary lymphoid organs. **(A)** Cells from the spleen: 1. F4/80^+^ macrophages. 2. NKp46^+^ cells. 3. T CD3^+^. 4. CD4^+^ T helper lymphocytes. 5. CD4^+^/Foxp3^+^ T lymphocytes. 6. CD8^+^ T cytotoxic lymphocytes. 7. CD8^+^/Foxp3^+^ T lymphocytes. **(B)** Immune cells from NLPs: 1. F4/80^+^ macrophages. 2. NKp46^+^ cells. 3. T CD3^+^. 4. CD4^+^ T helper lymphocytes. 5. CD4^+^/Foxp3^+^ T lymphocytes. 6. CD8^+^ T cytotoxic lymphocytes. 7. CD8^+^/Foxp3^+^ T lymphocytes. **(B)** Graphs represent the mean ± SD of the data from two independent experiments (4T1, *n* = 10; 4T1+*T. canis, n* = 10). Gate from 10,000 cells was collected. Statistical significance was calculated using a t-test (*p ≤ 0.05).

As mentioned before, *T. canis* infection modifies the systemic immune response in secondary lymphoid organs. In order to assess whether these modifications in splenic and lymph node immune cell proportions are present in infected tumor-bearing mice, innate and adaptive immune cells were analyzed. The splenic innate and adaptive cellular compositions were similar in the two groups (Figure 5).

Although no changes were observed in splenic immune cells (Figure 5A1-7), in PLNs from infected mice, the CD4^+^Foxp3^+^ lymphocyte proportion was increased (*p* = 0.0162) compared to the 4T1 control group (Figure 5B.5). Although the increase in CD8^+^ T cells from PLN was linked to the *T. canis* infection (28), when the tumor was induced, the CD8^+^ T lymphocyte proportion in PLNs was decreased (*p* = 0.0137) in comparison to the 4T1 control group (Figure 5B.6).

**Figure 6:**
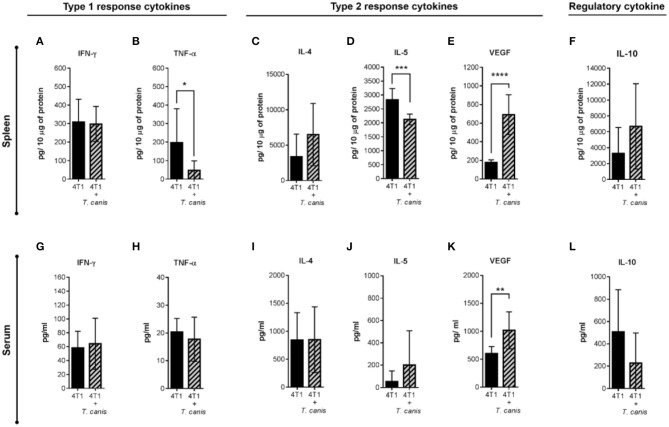
Analysis of systemic soluble factor expression in spleen and serum. Determination by sandwich ELISA of the splenic type 1 response cytokines. **(A)** IFN-γ. **(B)** TNF-α; the type 2 cytokines: **(C)** IL-4, **(D)** IL-5, **(E)** VEGF; and the regulatory cytokine. **(F)** IL-10. Analysis of soluble factors in serum. **(G)** IFN-γ. **(H)** TNF-α. **(I)** IL-4. **(J)** IL-5. **(K)** VEGF. **(L)** IL-10. Graphs indicate data from splenic proteins and sera obtained from two independent experiments (4T1, *n* = 10; 4T1+*T. canis, n* = 10). Bars represent the mean ± SD of cytokine levels (pg/10 μg of splenic proteins or pg/ml of sera). Statistical significance was calculated using a *t*-test (*p ≤ 0.05; **p ≤ 0.01; ***p ≤ 0.001; ****p < 0.0001).

Expression of systemic soluble factors was performed to determine whether the changes in the tumor microenvironment were related to the systemic response (Figures 6A–L). In the spleen, type 1 response cytokine TNF-α was decreased (*p* = 0.0311) in infected mice from the 4T1+*T. canis* group (Figure 6B). Although the type 2 cytokine IL-4 and the regulatory IL-10 (Figure 6C,F) were augmented in the spleen, these differences were not statistically significant. However, there was a reduction in splenic IL-5 (*p* = 0.0002) and a higher amount of VEGF (*p* < 0.0001) (Figures 6D,E).

Soluble factors measured in sera (Figures 6G–L) were mostly similar in the 4T1 and 4T1+*T.canis* groups, with the exception of an increased level of VEGF (*p* = 0.0030) in 4T1+*T. canis* mice (Figure 6K).

## Discussion

For many years, cancer research has been focused on a cell-central approach based on cancer cell signaling pathways and DNA changes (32), with the main goal of finding a cancer cure. This type of research has made some advances in cancer cell biology and treatment, but it has also neglected some cancer risk factors related to the interactions among cells, the tumor microenvironment, and infectious agents. Besides cell-cell interactions, the interaction with infectious agents is another important factor to consider in this sense. In particular, helminths are well-known as regulators of the host immune response through the generation of AAMs, Tregs, and B regulatory cells, mediated by helminth SE products (29).

In our study, we observed the effect of *T. canis* infection in the development of 4T1 mammary tumor model in BALB/c mice because we do not need any modification to the immune system of these animals to allow tumor growth and so we were able to assess the local and systemic reactions. Notably, after 28 days of 4T1 cell inoculation, enhanced tumor promotion was observed in *T. canis-*infected mice. Furthermore, the tumor microenvironment was modified, explaining the larger tumor size.

There is evidence that some helminths are inductors of different types of tumors (8), and the coexistence of diseases caused by helminths and enhancement of tumor growth has also been described. In a tumor model of colitis-associated colon cancer in mice, an intestinal infection with the nematode *H. polygyrus* promoted tumor growth (9). In another colon cancer model, the intestinal infection with the nematode *Trichuris muris* accelerated the progress of spontaneously developed intestinal adenomas in APC min/+ mice (33). In both studies, the nematode and the tumor are in the same anatomical location, but, *T. canis* larvae migrate through different organs (24), and the regulatory immune response induced by the parasite is widespread throughout different body compartments, which, in the 4T1 model, promotes the size of the mammary tumor.

Contrary to our initial hypothesis, tumor enlargement was not associated with an increase in the local proportion of T regulatory cells (CD4^+^/Foxp3^+^ and/or CD8^+^/Foxp3^+^). Although the tumor Treg proportion was not increased due to the infection in the PLN from infected mice, we detected a higher proportion of CD4^+^/Foxp3^+^ Treg cells in the PLNs of both *T. canis*-infected mice and the 4T1+*T. canis* group. Treg induction in tumors may be less frequent than Treg production in tumor-draining lymph nodes (TDLNs), perhaps because the conversion is more active in lymph nodes than in tumors (34).

The microenvironment in TDLNs is important in the progression of the tumor immune response (35). For example, Foxp3^+^ Treg cells in TDLNs modulate T lymphocyte function by suppressing IFN-γ secretion in CD8^+^ cells (35). Another mechanism by which Treg cells regulate CD8^+^ T lymphocyte expansion and modify differentiation is by competing for IL-2; at the same time, this cytokine may promote Treg functions (36). This may explain the increased proportion of CD4^+^/Foxp3^+^ Treg in PLNs, accompanied by a decreased proportion of CD8^+^ T lymphocytes in PLNs and the tumor infiltrate, from *T. canis* infected mice.

CD8^+^ anti-tumor function depends on its differentiation and infiltration into the tumor microenvironment (37). Differentiation progresses to CD8^+^ T cell cytotoxic function, which may induce tumor cell apoptosis through IFN-γ secretion (38). Although, in the present study, the percentage of CD8^+^ T cells was lower in tumors from 4T1+*T. canis* mice, the IFN-γ levels were unchanged with respect to non-infected mice. This may be because other hematopoietic cells, such as NK and CD4^+^ T cells, also produce IFN-γ within the tumor (39). The larger tumor size in the *T. canis* group could be related to the reduction in CD8^+^ T cell infiltration into the tumor, associated with deficient local recruitment. There could also be an impairment in the cytotoxic function of the recruited CD8^+^ cells. As mentioned previously, Treg cells play an important role in the suppression of IFN-γ secretion by CD8^+^ lymphocytes (35), but in our study, the Treg percentage was inferior in tumors of *T. canis-*infected mice. Therefore, Treg may not exert an important effect in CD8^+^ cell recruitment and function, but another population such as TAMs, through IL-10 secretion or tumor cell direct contact, could promote CD8^+^ cell impairment (40).

A Type 1 response linked to cell-mediated immunity usually prevents tumor growth because of a CTL response (41). Type 2 polarization promotes tumor development through the regulation of the host immune response and is associated with solid tumors (41, 42). This polarization is associated partially with cytokines in the tumor milieu (43), so we determined Type 1 and Type 2 cytokine expression of tumor extracts. Among the cells that promote the secretion of type 2 cytokines in the tumor microenvironment are the TAMs (42). This Type 2 polarization has been described in 4T1 BALB/c mouse tumors and is characterized by the presence of IL-4, VEGF, F4/80^+^ macrophages, and CD4^+^/Foxp3^+^ Treg cells (43). In the present study, these cytokines and cells were present in the 4T1 tumors, and in the 4T1+*T. canis* group, the cytokine milieu was enriched by the increase of IL-4, VEGF, and IL-10 and the decreased amount of the Type 1 cytokine TNF-α.

This microenvironment suggests that the intra-tumoral cell populations may be polarized as a Type 2 and/or regulatory phenotype. Thus, although we need to further characterize the F4/80^+^ macrophages and CD19^+^ B lymphocytes phenotype, these cells possibly display local Type 2 and regulatory functions. This polarization is reported in an *in vitro* experiment where macrophages obtained from *T. canis*-infected mice secreted higher amounts of IL-10 and lower quantities of TNF-α as compared to macrophages from uninfected mice (44). Furthermore, splenic Breg lymphocytes (CD19^+^) also secrete IL-10, which suppresses CD4^+^ and CD8^+^ T cells (45).

Type 2 response, solid tumor growth, and angiogenesis are also related (46). Angiogenesis plays an important role in tumor development and the spread of tumor cells through blood vessels (47), and as mentioned before, VEGF promotes angiogenesis (17). Thus, systemic and local VEGF increase in tumors from *T. canis*-infected mice is associated with the Type 2 response in the tumor microenvironment and promotes tumor growth.

The humoral response is related to *T. canis* infection, and antibody detection is the basis for immunological diagnostic tests (18). In the present study,the level of IgG at 49 d.p.i was very similar to that in *T. canis*-infected mice with tumors and B lymphocyte expansion in the spleen was also found (28). Thus, the systemic response to infection was evidenced by the specific humoral response.

In regard to splenomegaly, *T. canis* infection induced spleen enlargement (48), but also, the 4T1 tumor induction increased the spleen size (49) and induced splenic Treg depletion (3). Both abnormalities are associated with a leukemoid response and extramedullary (splenic) hematopoiesis in 4T1 tumor-bearing mice, in which an increased proportion of granulocytes diminishes the proportion of lymphocytes (50). This response may mask the expected modifications in immune cell proportions in the secondary lymphoid organs from the 4T1+T. canis mice.

Although IL-5 serum levels in infected tumor-bearing mice were not increased enough to be statistically significant, we observed an increase in this cytokine associated with the infection. Splenic IL-5 level in 4T1+*T. canis* animals were very similar compared to infected mice without tumor (28).

**Figure 7:**
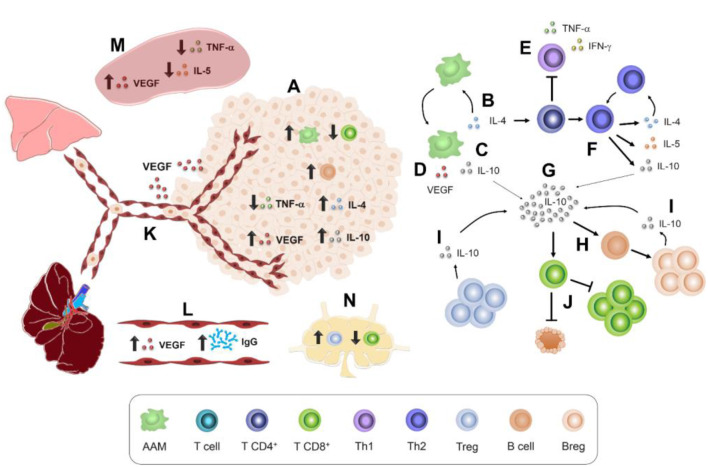
Immune interactions in 4T1 tumor-bearing mice chronically infected with *T. canis*. **(A)** Tumor immune microenvironment. AAM secretion of: **(B)** IL-4. **(C)** IL-10. **(D)** VEGF. **(E)** Th1 polarization inhibition. **(F)** Th2 polarization promotion by IL-4. **(G)** IL-10 production and stimulus in different immune cells. **(H)** B lymphocyte conversion to Breg lymphocytes. **(I)** IL-10-secretory Breg cells. **(J)** Inhibitory effect of IL-10 on T CD8^+^ proliferation and cytotoxic activity. **(K)** VEGF promotes angiogenesis and pulmonary and liver metastasis. **(L)** Higher levels of anti-*T. canis* IgG and VEGF in serum. **(M)** Splenic immune microenvironment. **(N)** PLN immune cell milieu.

Higher proportions of immune cells found in the tumor microenvironment (Figure 7A) were F4/80^+^ macrophages and CD19^+^ B cells, which could contribute to tumor enlargement. For example, macrophages such as AAMs produce IL-4, IL-10, and VEGF (15) (Figures 7B–D). These soluble factors act on other cell populations. For instance, IL-4 (Figure 7B) may inhibit Th1 polarization and, therefore, the production of TNF-α and IFN-γ (41) (Figure 7E), which stimulates CD8^+^ cytotoxicity (45). IL-4 also promotes Th2 polarization and thereby the secretion of IL-4, IL-10, and IL-5 (41) (Figure 7F), a typical cytokine of *T. canis* systemic immune response (28). Another AAM related-cytokine is IL-10 (15) (Figure 7G), which stimulates LB to Breg switch (51) (Figure 7H). Together, Treg and Breg produce and enrich an IL-10 milieu (Figure 7I). IL-10 inhibits cytotoxicity activity and proliferation of LT CD8^+^ cells (52) (Figure 7J). Meanwhile, local increase of VEGF (Figure 7A) enhances the blood vessel formation that nourishes the tumor and promotes metastasis to distant organs such as lungs and liver (17) (Figure 7K). This tumor VEGF was accompanied by systemic augmentation levels of this soluble factor in serum (Figure 7L) and spleen (Figure 7M). Additionally, CD8^+^ T cytotoxic cells may eliminate tumor cells (38), and a reduced proportion within the tumor in *T. canis*-infected mice could allow a larger tumor size (Figure 7A). Together with a lower CD8^+^ cell percentage in tumor, in PLN, the decrease in this population in infected animals could be associated with the increased proportion of Treg (36) (Figure 7N). On a systemic level, higher serum *T. canis*-IgG levels were sustained in tumor-bearing mice (Figure 7L). With regard to splenic changes associated with *T. canis* infection, TNF-α and IL-5 decrease were detected (Figure 7M).

This study showed that immune system modulation caused by *T. canis* infection leads to a different anti-tumor response and triggers tumor growth. This modulation was mainly associated with a tumor microenvironment characterized by a Type 2, regulatory immune, and angiogenic milieu.

Augmented tumor growth associated with *T. canis* infection is the result of complex interactions among the immune system, tumor cells, and the nematode larvae. In this intricate network, the immune response must act against two different etiologies that usually occur in everyday life and in all kinds of organisms. Therefore, the identification of risk factors that promote tumor progression by regulating the immune response is important for making decisions about lifestyle options and seeking medical attention. In this sense, *T. canis* infection prevention should be an important issue not only for the clinical disease itself but also because of an increased susceptibility to develop larger mammary tumors. Prevention is essential given the limited treatment efficacy against *T. canis* larvae encapsulated in tissues (27). Nevertheless, in human breast cancer patients or even in companion animals such as dogs and cats with mammary tumors, the screening of anti-*T. canis* antibodies to identify the infection is recommended to treat them and try to restrict the continuous promotion of a regulatory and angiogenic host immune response due to *T. canis*.

## Data Availability Statement

The data used in this study are available from the corresponding author upon reasonable request.

## Ethics Statement

The protocol used in this study was approved by the Committee on Ethics and Use in Animal Experimentation of the Instituto de Investigaciones Biomédicas, UNAM. The study was performed following the guidelines of Mexican regulations (NOM-062-ZOO-1999) and the Guide for the Care and Use of Laboratory Animals of the National Institute of Health, 8th Edition to ensure compliance with the established international regulations and guidelines.

## Author Contributions

JM-M, conceived and designed the study. RR-M, MP-A, RH-C, and VD developed the methodology. JM-M and KN-C acquired the data (provided animals, provided facilities, etc.). RR-M, PO-S, SM-C, and JM-M analyzed and interpreted data. JM-M, RR-M, PO-S, KN-C, SM-C, and VD wrote and/or reviewed the manuscript. RR-M, MP-A, and KN-C provided administrative, technical, or material support (i.e., reporting or organizing data, constructing databases). JM-M supervised the study.

## Conflict of Interest

The authors declare that the research was conducted in the absence of any commercial or financial relationships that could be construed as a potential conflict of interest.
